# A Chinese Chan-based lifestyle intervention improves memory of older adults

**DOI:** 10.3389/fnagi.2014.00050

**Published:** 2014-03-26

**Authors:** Agnes S. Chan, Sophia L. Sze, Jean Woo, Ruby H. Yu

**Affiliations:** ^1^Department of Psychology, The Chinese University of Hong KongNew Territories, Hong Kong, China; ^2^Chanwuyi Research Center for Neuropsychological Well-Being, The Chinese University of Hong KongNew Territories, Hong Kong, China; ^3^Henan Songshan Research Institute for ChanwuyiZheng Zhou, China; ^4^Department of Medicine and Therapeutics, The Chinese University of Hong KongNew Territories, Hong Kong, China

**Keywords:** memory, lifestyle intervention, Chinese, elderly, mind–body exercise, diet

## Abstract

This study aims to explore the potential benefits of a Chinese *Chan-*based lifestyle intervention on enhancing memory in older people with lower memory function. Forty-four aged 60–83 adults with various level of memory ability participated in the study. Their memories (including verbal and visual components) were assessed before and after 3 months intervention. The intervention consisted of 12 sessions, with one 90 min session per week. The intervention involved components of adopting a special vegetarian diet, practicing a type of mind–body exercises, and learning self-realization. Elderly with lower memory function at the baseline (i.e., their performance on standardized memory tests was within 25th percentile) showed a significant memory improvement after the intervention. Their verbal and visual memory performance has showed 50 and 49% enhancement, respectively. In addition, their improvement can be considered as a reliable and clinically significant change as reflected by their significant pre–post differences and reliable change indices. Such robust treatment effect was found to be specific to memory functions, but less influencing on the other cognitive functions. These preliminary encouraging results have shed some light on the potential applicability of the Chinese *Chan-*based lifestyle intervention as a method for enhancing memory in the elderly population.

## INTRODUCTION

Mild cognitive impairment (MCI), also known as isolated memory impairment, is a brain disorder primarily affects memory. While the older adults may begin to forget important information such as appointments or conversation, this impairment does not interfere with their daily activities (Petersen et al., 1999). Empirical evidence has showed that MCI is a risk factor for later progression to dementia. That is, studies have suggested that these individuals tend to progress to probable Alzheimer’s disease (AD) at a rate of approximately 10–15% per year (Bowen et al., 1997; Petersen et al., 1999, 2001). Scientific reviews have suggested that various cognitive-based interventions for AD type of dementia are rather labor intensive, yet with limited efficacy, particularly in managing the core symptom of memory deficits (Clare and Woods, 2004; Choi and Twamley, 2013). Therefore, some research efforts have been directed to explore effective preventive measures for normal elderly so as to reduce their risk of developing dementia. Because memory impairment is a relatively important pre-clinical feature of dementia, vigorous research efforts have focused on exploring methods to delay the decline or enhance the memory function in normal elderly (Rasmunsson et al., 1999; Mahncke et al., 2006; Papp et al., 2009; Smith et al., 2009; McAvinue et al., 2013). Nevertheless, as suggested by a recent review (Papp et al., 2009), the efficacy of various cognitive interventions for healthy elderly is rather limited. Some interventions have no significant effect (Rasmunsson et al., 1999; Edwards et al., 2002); others showed very minimal memory enhancement of <10% (at small effect size; Mahncke et al., 2006; Willis et al., 2006; McAvinue et al., 2013). For instance, Smith et al. (2009) reported results of an intensive computerized training (five 1 h sessions per week for 8 weeks) with approximately 10% post-intervention increment in delayed recall performance of elderly subjects.

Besides cognitive training, another emerging line of research has proposed lifestyle intervention for mental and physical wellness of the elderly. Lifestyle intervention, an integrative intervention comprising diet, exercise, and at least one other component (e.g., stress management, counseling, or smoking cessation), aims to reverse pathology and/or delay disease progression by modifying high-risk health habits (Lifestyle Medicine, 2013). Some research findings have supported the effects of certain lifestyle factors or some comprehensive lifestyle intervention programs in improving mental (e.g., depressive mood), and physical wellness (e.g., blood pressure level) of aging people (Chan et al., 2005; Dickinson et al., 2006; Witte et al., 2009; Ruscheweyh et al., 2011; Clark et al., 2012; McGregor et al., 2013). Nevertheless, the effect of lifestyle intervention on memory problem is relatively unclear.

Given the potential efficacy and easy accessibility of lifestyle intervention, this study aimed to explore the effect of a Chinese *Chan*-based lifestyle intervention, the* Dejian* Mind-Body Intervention (DMBI; Chan, 2010, 2013), on improving the memory function of community-dwelling elderly people. This intervention was developed upon the medical principles originating from the *Shaolin* Temple. DMBI, a holistic approach facilitating better well-being and quality of life through modification of one’s life habits, comprises diet modification, mind–body exercises, and self-realization. Cumulative empirical and clinical evidence has supported the positive effects of DMBI on cognitive functions, mood, problem behaviors, and physical health of healthy adults and patients with various brain disorders (Chan et al., 2008, 2009, 2011a,b,c, 2012a,b,c, 2013a,b, 2014). A randomized controlled study has found significantly improved attention and depressive mood (Chan et al., 2012a), and physical condition and sleep problem (Chan et al., 2012b) in patients with depression. Their enhancement in cognitive functions was associated with their altered brain electrophysiological activity pattern (Chan et al., 2013b). Other studies also showed improvement on various cognitive functions including attention, memory, self-control, and executive functions on children with autism (Chan et al., 2008, 2011a, 2012c, 2013a, 2014). Furthermore, some pilot data have suggested positive effects of DMBI on enhancing psychological and physical wellness of community-dwelling elderly (Yu et al., in press). Given the encouraging results on individuals with depression and autism and some preliminary evidence for elderly people, the present study extends the previous findings and examine if this intervention method can have positive effect on older adults with lower level of memory function. A group of independently living elders was recruited, and about 30% of them demonstrated certain degree of memory problems as suggested by their relatively poorer performance (at or below 25th percentile) on the standardized memory tests. The primary purpose of the present study is to examine if a Chinese *Chan*-based intervention can improve the memory function of this group of older adults.

## MATERIALS AND METHODS

### PARTICIPANTS

Forty-four participants aged 60–83 were recruited from six community health and social centers in the New Territories East regions in Hong Kong and through Internet publicity. Participants with a history of head injury or neurological/psychiatric disorders, who could not walk, or had severe illnesses, were excluded. They were blind to the potential benefits of the intervention. The attrition rate was 5%, and the mean attendance rate was 93%. The participants were divided into three groups according to their performance on the standardized verbal and visual memory tests: (1) high performers: score at or above 75th percentile; (2) medium performers: score between 26th and 74th percentile; (3) low performers: score at or below 25th percentile. The Hong Kong List Learning Test (HKLLT) and the Visual Reproduction subtest of the Wechsler Memory Scale III (WMS-VR) are selected as the tests for verbal and visual memory function and the classification of the three groups of performers are based on the performance of delayed recall in these two memory tests. The reason for choosing delayed recall performance is that it bears 90% accuracy in predicting the progression from MCI to dementia after 2 years (Tierney et al., 1996). In particular, the delayed recall in verbal learning test is one of the neuropsychological assessment indices that commonly used to measure memory decline associated with aging and MCI (Hulette et al., 1998; Artero et al., 2003; Gauthier et al., 2006) and the most accurate cognitive measure in discriminating between individuals with presymptomatic AD and those who remained without dementia (Chen et al., 2000).

**Table 1** presents the demographic characteristics of the three groups for the verbal and visual memory function. The global cognitive function of each participant was assessed by the Chinese version of Mattis Dementia Rating Scale (CDRS; Chan et al., 2003a) with a purpose to screen out participant having dementia. As revealed by their performance on the CDRS, all participants scored above the optimal cutoff score of the adjusted total score (i.e., 141), which suggested that none of them had dementia and they demonstrated intact global cognitive functions. In general, all three groups of performers had comparable age, education, gender, handedness, CDRS adjusted score, blood pressure level, and depressive mood problems (as measured by the Chinese Geriatric Depression Scale-short form (CGDS-SF; Lee et al., 1993), *F* ranges from 0.07–3.05, *p* > 0.05, except for the groups of visual memory function that the high performers were younger than the medium performers, *p* = 0.032, the low performers had lower CDRS and CGDS-SF score than medium performers, *p* = 0.006 and 0.027, and the low performers had higher systolic blood pressure level than high performers, *p* = 0.019.

**Table 1:** Demographic characteristics of participants.

Characteristics	Verbal memory performance					Visual memory performance				
	LP (*n* = 18)	MP (*n* = 9)	HP (*n* = 14)	*F* or χ^2^	*p*	LP (*n* = 10)	MP (*n* = 21)	HP (*n* = 11)	*F* or χ^2^	*p*
Age (years)	65.94 (5.54)	66.56 (5.81)	64.00 (2.86)	0.95	0.40	65.50 (4.84)	66.76 (5.37)	62.27 (1.90)	3.46	0.04*
Education (years)	4.28 (1.90)	4.11 (1.27)	4.79 (1.72)	0.52	0.60	3.50 (0.97)	4.43 (1.63)	5.18 (1.99)	2.86	0.07
Gender (male:female)	12:6	5:4	5:9	3.05	0.22	5:5	12:9	6:5	0.14	0.93
Handedness (right-handed %)	16:2	9:0	13:1	1.09	0.58	10:0	20:1	9:2	2.97	0.23
CDRS (adjusted total score)	165.64 (4.75)	168.86 (4.74)	167.52 (3.33)	2.28	0.12	163.76 (5.76)	168.74 (3.49)	166.60 (2.52)	4.83	0.01*
Systolic blood pressure	149.33 (20.39)	146.06 (10.35)	137.36 (17.66)	1.83	0.17	155.25 (14.75)	146.79 (19.35)	133.68 (15.48)	4.15	0.02*
Diastolic blood pressure	83.69 (9.69)	77.83 (12.52)	77.93 (8.09)	1.75	0.19	83.85 (12.21)	80.69 (11.33)	79.00 (7.41)	0.56	0.58
CGDS-SF	2.39 (3.11)	2.00 (1.80)	2.29 (2.27)	0.07	0.94	4.10 (3.54)	1.67 (1.59)	1.82 (2.27)	3.93	0.03*

LP = Low Performers who score at or below 25th percentile rank on the verbal or visual memory test; MP = medium performers who score at 26th–74th percentile rank on the verbal or visual memory test; HP = high performers who score at or above 75th percentile rank on the verbal and visual memory test; CDRS = Age- and education-adjusted total score of the Chinese version of Mattis Dementia Rating Scale; CGDS-SF = Total score of the Chinese Geriatric Depression Scale-short form. *p < 0.05.

### PROCEDURE

The study was conducted in accordance with the Helsinki Declaration of the World Medical Association Assembly. The research protocol was approved by the Joint Chinese University of Hong Kong – New Territories East Cluster Clinical Research Ethics Committee. All participants voluntarily participated in the study, and their informed consent was obtained prior to the study. Research assistants who were blind to the experimental design and potential effects of the intervention individually assessed the demographic information, medical history, and cognitive functions of each participant before and after intervention. After the baseline assessment, the participants attended group training on the Chinese *Chan*-based lifestyle intervention for 12 weekly 90 min sessions.

### INTERVENTION

The DMBI is a Chinese *Chan*-based lifestyle intervention based on the *Chanwuyi* tradition (i.e., Zen, martial arts and healing) and the *Chan* medical principle. According to the *Chan* medical principle, majority of mental and physical health problems are due to the blockage of orifices (i.e., bodily openings) and stagnation of *Qi* and blood circulation, which are likely resulted from unhealthy living style and attitude. Thus, adoption of a healthy lifestyle and positive thinking is a key to unblock the orifices and smooth the *Qi* and blood circulation, so as to enhance health. Emphasizing integrative treatment of the mind and the body, the principle of DMBI is to enhance mental and physical health by changing daily dietary and exercise habits and to improve the psychological well-being by understanding the root of problems in accordance with Buddhist philosophy (Chan, 2010, 2013).

The intervention was conducted by a clinical psychologist who is familiar with the DMBI model. Throughout the 12 training sessions, the participants were taught the fundamental principles and techniques of DMBI, and their progress was closely monitored by the trainer. There were three intervention components: (1) Advise diet modification by reducing intake of food (e.g., ginger, garlic, green onions, spicy foods, eggs, meat, and fish) that generates excessive internal heat and adversely affects moods and physical health based on *Chan* medical principle; and consuming food that was good for health everyday (e.g., fresh vegetables, fruits, grains, beans, mushrooms, nuts, and root vegetables). (2) Practice *Nei Gong,* which is mind–body exercise, composing sets of breathing exercises and gentle and calm movement. For instance, rolling the hands slowly up and down between the chest and the abdomen, resting the hands on the abdomen while quietly observing the breathing. Regular practice of *Nei Gong* helps reducing stress, and improving overall physical health and the circulation of *Qi* and the blood. The basic principles and demonstration of *Nei Gong* have been elaborated on our website (www.chanwuyicenter.com) and in two published books (Chan, 2010, 2013). The practice time was not fixed, and participants were instructed to practice the exercises until they felt warm and relaxed. (3) Improving psychological well-being by increasing awareness and sensitivity to how unrealistic desires (i.e., greed), anger, and obsession (i.e., unrealistic craving for something or somebody) affect mental and physical health, and modifying thought process to alleviate excessive desires, anger, and obsessions.

According to the log record of home practice, 60% participants abstained from or reduced their intake of the “not recommended” food, and 81% participants consumed at least three “recommended” food categories every day. Also, 95% participants practiced *Nei Gong* every day for an average of about 25 min per day.

### MEASURES

#### Hong Kong List Learning Test

The HKLLT is a well-standardized test of verbal memory in the Chinese population (Chan et al., 2003b; Chan, 2006). Participants were required to learn a list of 16 Chinese words through three learning trials. After 30 min delay, they were asked to recall as many words as possible. The total number of recalled words after a delay was used to reflect verbal memory ability.

#### Visual reproduction subtest of the Wechsler Memory Scale III

The VR is a common test for visual memory function (Wechsler, 2005). Participants were required to learn five sets of geometric forms, and then draw them from memory after 30 min. The total score in terms of accuracy rate at a delayed recall was adopted for analyses. In addition, the total score at Copy trial, which simply required the participant to copy the geometric forms on the paper, was used to reflect the ability to construct visual forms, and to control the possible influence of deficient drawing ability on recall performance. It should be noted that the lowest Copy score in present study is 90 out of 104 (accuracy rate = 87%), which means that all participants have intact drawing ability and thus poor recall performance, if any, is unlikely due to deficient drawing ability.

#### Chinese Version of Mattis Dementia Rating Scale

The CDRS is a locally validated test that was culturally adapted from the Mattis Dementia Rating Scale to measure general cognitive functions, as indicated by its education- and age-adjusted total score, of the elderly people (Chan et al., 2001, 2003a). This scale was found to have good reliability with internal consistency ranging from 0.7–0.9, and satisfactory construct validity (Chan et al., 2001). It consists of 36 tasks in five subscales, including Attention, Memory, Conceptualization, Initiation/Perseveration (CDRS-I/P), and Construction, which tap abilities of visual and auditory attention and memory, abstraction, flexible cognitive control and visuospatial construction, respectively. The maximum total raw score of the scale is 144. However, the adjusted total score, instead of the total raw score, is adopted so as to control the confounding effect of age and education. Adjusted total score that is below the optimal cutoff score of 141 suggests higher probability of having dementia.

#### Color Trail Test

The Color Trail Test (CTT) is a standardized test for testing flexible set-shifting behavior (D’Elia et al., 1996). The total time (s) to complete the second trial of the CTT (CTT-T2), which requires connecting numbers in correct sequence while alternating between two colors embedding the numbers, was used to reflect degree of flexibility in mental shift.

#### Category Fluency Test

The Category Fluency Test (CFT) is a test for language production and fluency of speech (Chan and Poon, 1999). It requires generation of exemplars of two semantic categories, namely animals and transportation, as many as possible within time constraint. The total number of items generated was used to measure degree of fluent speech production.

### STATISTICAL ANALYSIS

The statistical package for the social sciences (SPSS; version 15.0) was used for data analysis. Repeated measures of analysis of variance (ANOVA) and *post hoc* paired *t-*tests were used to evaluate the changes across the three groups of performers. The extent of pre–post difference was evaluated with effect size. Additionally, the reliable change index (RCI) was computed to examine the clinical significance and reliability of the intervention effect. The RCI is calculated by dividing the pre–post difference score by the standard error of difference between the two test scores $\left(RCI=\frac{x2‑x1}{Sdiff}\right)$ (Jacobson and Truax, 1991). As proposed by Wise (2004), a clinically significant and reliable change can be reflected by a pre–post difference scores of >0.5 SD and a RCI > 0.84. χ^2^ statistics was adopted to compare number of participants with or without a clinically significant and reliable change in memory performance.

## RESULTS

### GREATER ENHANCEMENT IN MEMORY FUNCTION OF LOW-PERFORMING ELDERLY AFTER INTERVENTION

**FIGURE 1:**
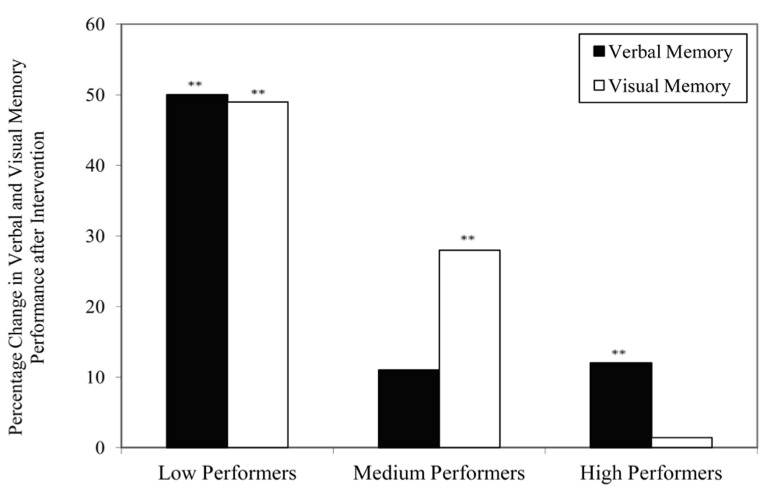
**Percentage change in raw scores of verbal and visual memory tests across three levels of performance subgroups after intervention.** **p < 0.01 in paired samples *t*-test (one-tailed).

Comparisons on the change in memory performance across three groups of elderly having different baseline levels of memory functioning revealed that elderly with lower performance level at baseline demonstrated greater extent of enhancement after intervention. Results of repeated measures ANOVA showed that there were significant Time (Pre vs. Post) by Group (three subgroups) interaction effect for both HKLLT, a measure of verbal memory, *F*(2,38) = 3.58,* p *= 0.038, and VR, a measure of visual memory, *F*(2,39) = 4.41, *p *= 0.019. **Table 2** presents the pre–post changes across the three groups analyzed by *post hoc* *t*-test. It was found that the low performers showed a significant improvement in both verbal, *t *= -6.89, *p *= 0.00, effect size = 1.62, and visual memory, *t *= -2.80, *p *= 0.01, effect size = 0.88, with an extent of 50 and 49% improvement, respectively (**Figure 1**). However, the extent of score-increment in medium and high performers was to a lesser extent (**Table 2**), which ranges from 1.4–28% (**Figure 1**). It should be noted that the extent of memory enhancement in low performers was significantly greater than that of the medium (HKLLT: *p *= 0.006) and high performers (HKLLT: *p *= 0.002; VR: *p *= 0.006).

**Table 2:** Memory performance of the three groups of performers at pre- and post-intervention.

	Pre	Post	Difference	*t* value	*p* value	E.S.
**Low performers**
Verbal memory (*n* = 18)	6.06 (1.26)	9.06 (1.80)	3.00 (1.85)	-6.89	0.00**	1.62++
Visual memory (*n* = 10)	35.10 (9.72)	52.40 (21.51)	17.30 (19.56)	-2.80	0.01*	0.88++
**Medium performers**
Verbal memory (*n* = 9)	8.89 (0.93)	9.89 (3.10)	1.00 (3.08)	-0.97	0.18	0.32
Visual memory (*n* = 21)	57.90 (8.68)	74.05 (9.91)	16.14 (12.99)	-5.70	0.00**	1.24++
**High performers**
Verbal memory (*n* = 14)	12.07 (1.07)	13.50 (1.51)	1.43 (1.60)	-3.33	0.00**	0.89++
Visual memory (*n* = 11)	79.45 (5.20)	80.55 (14.02)	1.09 (13.27)	-0.27	0.40	0.46

Low Performers = Subgroup of elderly having baseline level of performance on verbal and visual memory tests that falls at 25th percentile or below; medium performers = Subgroup of elderly having baseline level of performance on verbal and visual memory tests that falls within 26th–74th percentile; high performers = Subgroup of elderly having baseline level of performance on verbal and visual memory tests that falls at 75th percentile or above; Difference = Mean difference between scores at pre- and post-intervention; E.S. = Effect size. Standard deviations are in parenthesis. *p < 0.05, **p < 0.01 at one-tailed; ++large effect size.

**FIGURE 2:**
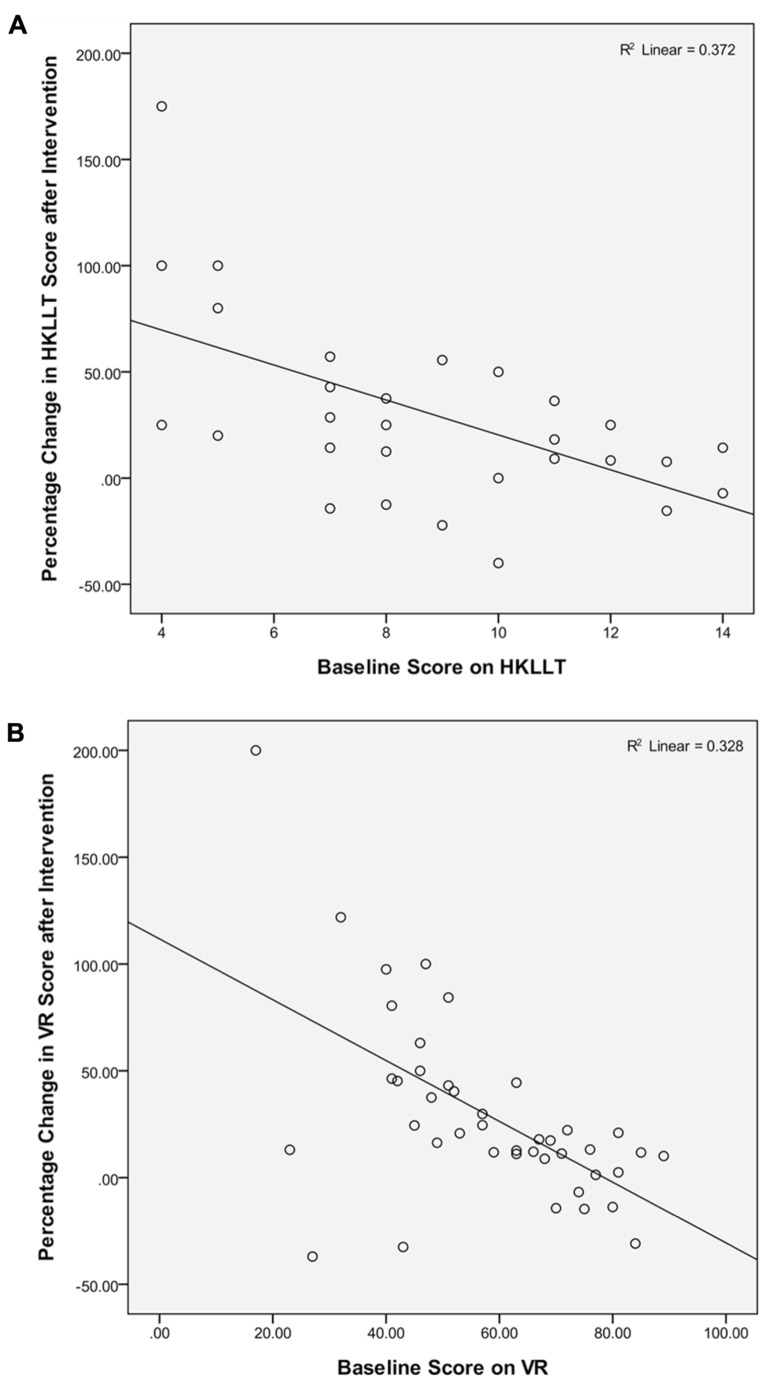
**Association between baseline level of performance on (A)** verbal and **(B)** visual memory tests and the percentage change in test performance after intervention.

The scatter-plots in **Figures 2A,B** showed a negative association between the baseline level of performance on verbal and visual memory and the percentage change in respective test performance after intervention, which was statistically significant, *r* = -0.61 and -0.57, *p* = 0.00. It suggests that elderly individuals with lower baseline level of memory function tend to demonstrate greater extent of memory enhancement after intervention, as compared to those having higher functioning level at baseline. These results implicate that the *Chan*-based lifestyle intervention seems to have positive effects on enhancing memory functions of older people who have relatively poorer memory.

### MEMORY ENHANCEMENT IN LOW PERFORMERS IS RELIABLE AND CLINICALLY SIGNIFICANT

In order to determine if the intervention effect on memory function is a reliable and clinically significant change, the Low Performers were further classified into either “Improved”, “No Change,” or “Declined” categories based on the criteria of pre–post change in test scores and RCI suggested by Wise (2004), which has been defined in **Table 3**. It was found that 72% participants demonstrated a clinically significant and reliable increase in verbal memory performance (i.e., “Improved” cluster), which was significantly more than the proportion of participants classified into the other two clusters, χ^2^(2) = 14.33, *p *< 0.001. A similar result was found for visual memory performance, where 60% participants demonstrated clinically significant memory enhancement, though such distribution did not reach statistical significance, χ^2^(2) = 3.80, *p *= 0.075. Therefore, the memory enhancement effect of DMBI for the elderly participants with a lower level of memory function was clinically significant and reliable. It suggests that the *Chan*-based lifestyle intervention might be an effective memory intervention for older people with poorer memory performance.

**Table 3:** Distribution of participants in the low performers with and without clinically significant changes in memory performance after intervention.

	*N*	χ^2^ (df)	*p* value
Verbal memory (*n* = 18)		14.33(2)	0.00**
Improved	13		
No Change	5		
Declined	0		
Visual memory (*n* = 10)		3.80 (2)	0.075
Improved	6		
No change	3		
Declined	1		

Improved = pre–post change in test scores > +0.5 SD and RCI > +0.84; No change = pre–post change in test scores in-between -0.5SD and +0.5SD and -0.84 < RCI < +0.84; Declined = pre–post change in test scores >-0.5 SD and RCI > -0.84; **p < 0.01 at one-tailed.

### SPECIFIC TREATMENT EFFECT ON MEMORY FUNCTIONS OF OLDER PEOPLE

To explore whether the positive treatment outcome was specifically for memory functions or the treatment would also benefit the other cognitive aspects of the older people, pre–post comparisons on the other cognitive functions based on the whole group was performed with paired samples *t*-tests. **Table 4** presents the performance of the participants on various cognitive tests before and after intervention. It was found that the most robust treatment effect was still in verbal and visual memory function. In the verbal memory test, there was a significant mean increment of two words recalled after intervention, *t* = -5.81, *p* = 0.00, effect size = 0.91, with an extent of 23% improvement. A similar degree of enhancement was found in visual memory test, which showed a significant 12-point increment in delayed recall score after intervention, *t* = -5.05, *p* = 0.00, effect size = 0.78, with an extent of 21% improvement. Although there was a significant improvement in the cognitive tests that measures ability to flexibly shifting mental set (CDRS-I/P and CTT-T2), *t* = -2.76 and 3.55, *p* = 0.01 and 0.00, respectively, their extent of improvement was to a much lesser degree (CDRS-I/P: 3%; CTT-T2: 12%). Furthermore, as expected, some other relatively more crystallized cognitive ability, such as language, and the cognitive abilities that are less susceptible to aging-related cognitive decline, such as visuospatial construction, did not show any significant changes after intervention, *t* ranges from -1.0 to 1.95, *p* > 0.05. In summary, the results suggested that the *Chan*-based lifestyle intervention seemed to have memory-specific positive effects for older people.

**Table 4:** Changes in various cognitive functions before and after intervention.

	Pre	Post	Difference	*t*	*p*	E.S.
**Memory**
HKLLT-DR	8.73 (2.89)	10.76 (2.85)	2.02	-5.81	0.00**	0.91++
WMS-VR-DR	58.12 (17.76)	70.60 (17.63)	12.48	-5.05	0.00**	0.78+
**Attention**
CDRS-Att	36.43 (1.17)	36.57 (0.67)	0.14	-0.78	0.22	0.12
**Cognitive flexibility**
CDRS-I/P	35.43 (2.48)	36.40 (1.15)	0.98	-2.76	0.00**	0.43
CTT-T2^#^	109.66 (40.39)	96.88 (37.99)	-12.78	3.55	0.00**	0.55+
**Conceptualization**
CDRS-concept	37.67 (1.98)	37.74 (1.95)	0.07	-0.24	0.41	0.04
**Language**
CFT	29.17 (5.21)	29.86 (4.42)	0.69	-0.93	0.18	0.14
**Visuospatial construction**
CDRS-construct	5.93 (0.26)	5.98 (0.15)	0.05	-1.0	0.16	0.15
WMS-VR-copy	98.90 (3.06)	98.19 (3.19)	-0.71	1.95	0.03*	0.30

HKLLT-DR = Delayed recall score of the Hong Kong List Learning Test; WMS-VR-DR = Delayed recall score in the Visual Reproduction subscale of the Wechsler Memory Scale-III; Chinese version of Mattis Dementia Rating Scale CDRS-Att = Attention subscale score of the CDRS-I/P = Initiation/Perseveration subscale score of the CDRS; CTT-T2 = Completion time of the Color Trail Test–Trial 2; CDRS-Concept = Conceptualization subscale score of the CDRS; CFT = Category Fluency Test; CDRS-Construct = Construction subscale score of the CDRS; WMS-VR-Copy = Copy score in the VR of the WMS-III; Difference = Post-intervention score minus pre-intervention score; E.S. = Effect size. Standard deviations are in parenthesis. ^#^Lower value indicates better performance; *p < 0.05, **p < 0.01; + medium effect size, ++large effect size.

## DISCUSSION

The findings of the present study provide preliminary support for the effects of a 3 months Chinese *Chan*-based lifestyle intervention in improving memory function of older adults with poorer memory performance. The extent of increment in delayed recall score (for 50%, at large effect size) on both verbal and visual memory in present study was very encouraging. Such improvement met the criteria of clinical significance and reliable change, suggesting that the improvement is likely treatment-specific and probably not due to practice effect. The previous studies on various cognitive training (Mahncke et al., 2006; Willis et al., 2006; Smith et al., 2009) and lifestyle intervention (Witte et al., 2009; Ruscheweyh et al., 2011) reported some positive effects on memory function that is consistent with the present study, suggesting that memory ability can be improved with appropriate intervention. However, the effects of the present intervention seem to be to a greater extent compared to the conventional cognitive training program and other lifestyle interventions. For instance, 3 month caloric restriction can trigger a 20% increment in delayed recall (Witte et al., 2009), 6 month low-intensity aerobic exercise training can induce a 10% increase in immediate recall (Ruscheweyh et al., 2011), and present results demonstrated a 50% improvement on delayed recall. It should also be noted that the present study recruited elderly with isolated memory difficulty while the other studies did not target older adults with memory difficulty. Thus, the memory-enhancing effect of the *Chan*-based lifestyle intervention has implicated its possibility as a method in facilitating memory function in older adults or even individuals with MCI.

The present result was consistent with our previous studies on other clinical populations (Chan et al., 2008, 2009, 2011a,b,c, 2012a,b,c, 2013a,b, 2014). In a series of studies on autism, the participants demonstrated significant improvement on cognitive function after 1 month intervention on changing diet (Chan et al., 2012c) and practicing *Nei Gong* (Chan et al., 2013a). In addition, an autistic child with moderately–severely impaired memory ability was found to have improved up to normal low-average range after an 8 month *Chan*-based intervention (Chan et al., 2011a). Similarly, randomized controlled studies on patients with clinical depression showed significant improvement on attention (Chan et al., 2012a) and brain activity pattern (Chan et al., 2013b). Thus, the results of the present study further suggest that this intervention is not only applicable to children and adults but also to elderly people. One reason for the generalization of this intervention is that it involves easy and concrete procedure, and thus even individuals with limited intelligence, and ability are able to follow.

The low attrition rate of 5% hinted that the Chinese *Chan*-based lifestyle intervention was very well received by the elderly Chinese participants. This intervention has several merits that can provide clues to this phenomenon. First, the intervention techniques are simple and clearly comprehensible to older adults, e.g., what to eat and what not to eat, and the simple and slow movements of the mind–body exercises. This characteristic is particularly important for the local elderly population, as majority of them are illiterate or have less than 12 years of formal education. Second, as a lifestyle intervention, the treatment components are more accessible and applicable to daily home practice. While the majority of conventional cognitive training methods involve computerized programs, the *Chan*-based lifestyle intervention requires no special equipment, and only needs limited space for practicing the mind–body exercises, which seems to be more practical and feasible for elderly people of lower socioeconomic status or with limited computer knowledge. Third, this intervention has relatively less restriction in treatment regimen as compared to some other intervention methods. The participants were only recommended to modify their diet and practice the mind–body exercises according to their own lifestyles and plans. The mind–body exercise practice time of the participants averaged 25 min per day (range 5–60 min), and it can be broken down into smaller periods for shorter practice (e.g., 10 min each time, thrice per day). Such flexibility was not physically demanding for the elderly participants. Finally, the intervention can be conducted on a large scale and requires no in-depth analysis of the personal background and psychological distress of the elderly participants. Therefore, older people, especially the more conservative and traditional Chinese elderly, would feel more comfortable in participating, and the intervention can be run in community centers or homes for the elderly.

Despite these encouraging findings, further verification of the treatment effects on elderly participants with a larger sample size in randomized controlled trials is warranted. Furthermore, the participants in present study are cognitively intact as revealed by their relatively high CDRS score and their memory functions are within normal range, whether the memory-enhancement effect can also be found for elderly people with significant memory deficits or even with dementia is worth investigating. This study focused mainly on the effects on memory function, its effect on the mental and physical domains of the aging population deserves further investigation. In addition, whether this intervention can be applied to non-Chinese population remains unclear and needs further investigation.

## Conflict of Interest Statement

The authors declare that the research was conducted in the absence of any commercial or financial relationships that could be construed as a potential conflict of interest.
